# Associations of body mass index with mortality in heart failure with preserved ejection fraction patients with ischemic versus non-ischemic etiology

**DOI:** 10.3389/fcvm.2022.966745

**Published:** 2022-08-04

**Authors:** Shan Zeng, Xingming Cai, Yuxiang Zheng, Xiao Liu, Min Ye

**Affiliations:** ^1^Key Laboratory of Prevention and Treatment of Cardiovascular and Cerebrovascular Diseases, Department of Cardiology, First Affiliated Hospital, Ministry of Education, Gannan Medical University, Ganzhou, China; ^2^Department of Geriatric, The First Affiliated Hospital of Sun Yat-sen University, Guangzhou, China; ^3^Second Clinical Medical College, Nanchang University, Nanchang, China; ^4^Department of Anesthesiology, National Cancer Center/National Clinical Research Center for Cancer/Cancer Hospital, Chinese Academy of Medical Sciences and Peking Union Medical College, Beijing, China; ^5^Department of Cardiology, Sun Yat-sen Memorial Hospital, Sun Yat-sen University, Guangzhou, China; ^6^Department of Cardiology, The First Affiliated Hospital of Sun Yat-sen University, Guangzhou, China

**Keywords:** HFpEF, etiology, body mass index, death, obesity paradox

## Abstract

**Background:**

Obesity could paradoxically improve prognosis in patients with heart failure (HF), termed the “obesity paradox.” Whether HF etiology could modify the “obesity paradox” is still controversial. In the present study, we aimed to assess the relationship between obesity and death in patients with heart failure with preserved ejection fraction (HFpEF) with non-ischemic versus ischemic etiologies.

**Methods:**

We analyzed 3,360 HFpEF patients from the TOPCAT (Treatment of Preserved Cardiac Function Heart Failure with an Aldosterone Antagonist) trial. Cox regression models were used to assess the association of obesity assessed by body mass index (BMI) with short-term and long-term death risk.

**Results:**

Overweight and obesity were associated with a lower risk of long-term all-cause death in patients with non-ischemic HFpEF, even in those with class III obesity (adjusted HR: 0.61, 95% CI 0.38–0.97). However, in the ischemic subgroup, as obesity advanced, this paradoxical relationship was gradually attenuated and disappeared in class III obesity (adjusted HR: 0.93, 95% CI 0.56–1.57). Restricted cubic spline analyses confirmed the differential relationship of baseline BMI with risk of long-term death with a BMI higher than 30 kg/m^2^ in non-ischemic versus ischemic HFpEF. In the short-term follow-up, the beneficial effects of overweight and obesity on survival were consistently observed in all the BMI categories, with the nadirs of all-cause death risk at class III obesity category both in non-ischemic and ischemic subgroups.

**Conclusion:**

“Obesity paradox” was evident both in non-ischemic and ischemic HFpEF during short-term follow-up, even in those with class III obesity. However, the beneficial effect of class III obesity disappeared during long-term follow-up in ischemic HFpEF.

**Clinical Trial Registration:**

[https://clinicaltrials.gov], identifier [NCT00094302].

## Introduction

Obesity is a well-established independent risk factor for developing cardiovascular (CV) diseases such as heart failure (HF) in the general population (1, 2). However, multiple large studies have demonstrated a better prognosis for overweight and mild-to-moderate obese [defined by body mass index (BMI)] patients who have already developed HF across the whole spectrum of left ventricular ejection fraction (LVEF) (3, 4). This counterintuitive phenomenon is termed the “obesity paradox.” Recently, several possible explanations of the “obesity paradox” in HF have been proposed. Greater metabolic reserves, less cachexia, and increased amount of lean mass in obese patients may be possible contributors to the prognosis benefit of obesity for HF (5, 6). Since the underpinning mechanisms are still not fully understood, the “obesity paradox” in HF has been proposed for many years but the applications to clinical practice are still debated.

In the last several years, the potential confounders of the obesity-related survival benefit in HF population have been broadly explored in different clinical conditions (e.g., age, gender, etiology of HF, diabetes, hypertension, and other comorbidities) (7). However, only a few limited studies have reported the impact of HF etiology on “obesity paradox” in HF population and demonstrated distinct findings (8–10). Especially, these prior studies included mainly patients with heart failure and reduced ejection fraction (HFrEF) or relatively small sample of patients with HF and preserved ejection fraction (HFpEF). In fact, although patients with HFpEF have similar rates of hospitalization and death to those with HFrEF, these two entities manifest heterogeneous pathophysiology and receive different therapeutic regimens (11). In addition, recently published results from the large registry (12) revealed differential associated pattern of obesity and mortality among HFpEF and HFrEF patients, which potentially suggests differing mechanistic factors. Hence, the findings about the role of HF etiology on obesity paradox in HFrEF population could not be directly generalized to HFpEF population, and the interplay between obesity and HF etiology on mortality in patients with HFpEF still remain unclear.

Based on data from the TOPCAT (Treatment of Preserved Cardiac Function Heart Failure with an Aldosterone Antagonist) trial, our current study sought to explore the relationship between obesity (defined by BMI) and death in a large cohort of HFpEF patients with non-ischemic versus ischemic etiologies.

## Materials and methods

### Study population

The present study used data from the TOPCAT trial. The selection criteria and study design of TOPCAT have been described in detail previously (13–15). Briefly, TOPCAT was a multicenter, international, randomized, double-blind, placebo-controlled trial that evaluated the effects of spironolactone versus placebo on clinical outcomes in patients with HFpEF. In total, 3,445 patients with symptomatic HFpEF from 270 sites in 6 countries were enrolled between August 2006 and January 2012. All participants provided written informed consent, and the study protocol was approved by the ethics committee by ethics committees or institutional review boards at each participating site. Eligible subjects were patients aged ≥50 years with symptomatic HF and a LVEF ≥45%, and with either elevated natriuretic peptide level within the previous 60 days or hospitalization for HF within the previous 12 months before randomization. Excluded criteria included: severe systemic illness with a life expectancy <3 years; severe chronic pulmonary disease; severe renal dysfunction; known infiltrative or hypertrophic obstructive cardiomyopathy or known pericardial constriction; heart transplant; or known chronic hepatic disease. For the current study, we further excluded patients with missing information regarding BMI and potential confounders, as well as BMI <18.5 kg/m^2^ (*n* = 85), resulting in a final sample size of 3,360.

Given the previously reported significant differences in population characteristics and outcomes by region observed in TOPCAT (16), a sensitivity analysis was performed in the subgroup of patients enrolled in the Americas (United States, Canada, Brazil, and Argentina, *N* = 1,767). Also, patients with missing information regarding BMI and potential confounders, as well as BMI <18.5 kg/m^2^ were excluded (*n* = 60), resulting in a final sample size of 1,707.

### Exposure definitions

Patients were classified according to HF etiology with either non-ischemic or ischemic HFpEF. Ischemic etiology was defined as the presence of a history of myocardial infarction or revascularization (10). BMI was calculated as the ratio between weight in kilograms (kg) and the square of height in meters (m^2^). In the present study, patients were categorized according to the World Health Organization BMI classifications: normal weight (18.5–24.9 kg/m^2^), overweight (25.0–29.9 kg/m^2^), Class I obesity (30.0–34.9 kg/m^2^), Class II obesity (35.0–39.9 kg/m^2^), and Class III obesity (≥40.0 kg/m^2^) (17).

### Outcomes

Outcomes in the TOPCAT trial were adjudicated by a clinical endpoint committee, as described previously (13). In our current study, the primary outcome of interest was all-cause death and the secondary outcome was CV death during the follow-up period. CV death included death from myocardial infarction, sudden death, pump failure, pulmonary embolism, stroke, and CV procedure-related events. All events were reported by the primary site investigator and independently adjudicated by a blinded clinical endpoint committee. Definitions of these endpoints have been previously published (13).

### Statistical analysis

Continuous variables were summarized as means with standard deviations if normally distributed or as median and interquartile range otherwise. Categorical variables were expressed as numbers and percentages. Baseline characteristics were compared using standard parametric or non-parametric methods as appropriate. Kaplan–Meier curves were constructed from the date of enrollment to the incidence of death according to baseline BMI category and compared using the log-rank test (using normal weight patients as reference) in patients with non-ischemic versus ischemic HFpEF. To explore the short-term and long-term effect of BMI on outcomes in HFpEF patients with non-ischemic versus ischemic etiology, we assess the relationship of death with baseline BMI, as well as time-updated BMI, which represented the most recent available BMI value for each patient at each visit during the course of the trial. First, Cox regression analyses were performed to calculate hazard ratios (HRs) and their confidence intervals (CIs) of baseline BMI category in HFpEF according to HF etiology. To flexibly evaluate the associations of the continuous variable of baseline BMI with death, restricted cubic spline analyses with three knots (at the 10th, 50th, and 90th percentiles) were performed using 30 kg/m^2^ as reference in patients with non-ischemic versus ischemic HFpEF. Second, we conducted Cox regression analyses with time-updated BMI to examine the short-term impact of BMI on outcomes in non-ischemic versus ischemic HFpEF (18). Because there were up to 16 trial visits and the interval between visits were up to 6 months in the program, BMI value was updated up to 15 times after baseline for each patient to demonstrate its short-term effect on outcomes in non-ischemic versus ischemic HFpEF.

The covariates in the Cox regression model included randomization arm (spironolactone or placebo), age, gender, race, region of enrollment (Russia/Georgia versus the Americas), New York Heart Association (NYHA) functional class, heart rate, systolic blood pressure (BP), diabetes, stroke, atrial fibrillation, and serum creatinine. These variables were selected because of their well-established prognostic significance as CV risk factors and previous prognostic implication in the TOPCAT. In time-updated Cox regression models, we controlled for time-updated heart rate, time-updated systolic blood pressure and time-updated NYHA functional class at each visit instead of the baseline ones. We also adjusted for weight change during follow-up when modeling for baseline BMI; whereas, we replaced weight change with baseline BMI when modeling for time-updated BMI. In multivariate Cox regression models, we first fit a cox regression model with all covariates and used stepwise selection with the AIC method to produce a final set of potential confounders. This selection process was performed to produce a global model and then plus BMI levels to construct the final Cox proportional regression models.

Statistical analyses were performed using R version 4.0.3 software (Vienna, Austria). A two-tailed *p*-value of <0.05 was considered statistical significance.

## Results

### Baseline patient characteristics

The baseline characteristics of the study group subdivided according to HF etiology are presented in Table 1. The mean age of the overall cohort at baseline was 68.5 ± 9.6 years, and 1,629 (48.5%) were males. A total of 1,214 (36.1%) patients had HFpEF of ischemic etiology. The non-ischemic HFpEF subgroup was comprised of more females but fewer White and smokers, as well as smaller waist circumstance, as compared to the ischemic subgroup. Besides, patients with non-ischemic HFpEF had higher blood pressure, heart rate, LVEF, and better NYHA functional class than those with ischemic HFpEF. With respect to comorbidities, the non-ischemic subgroup had a significantly lower prevalence of peripheral arterial disease, dyslipidemia, diabetes mellitus (DM), but a higher rate of atrial fibrillation (AF), compared with the ischemic subgroup. As for cardiac medications, patients with ischemic HF received more β-blocker, statin, and aspirin therapy than those with non-ischemic HF. Patients with non-ischemic HF received more warfarin therapy, in coincidence with the higher prevalence of AF in this subgroup.

**TABLE 1:** Patient characteristics according to heart failure etiology.

Variable	Overall (*N* = 3,360)	Heart failure etiology		*P*-value*
				
		Non-ischemic heart failure (*N* = 2,146)	Ischemic heart failure (*N* = 1,214)	
Randomization to spironolactone, *n* (%)	1,687 (50.2)	1,084 (50.5)	603 (49.7)	0.665
Age, years	68.5 ± 9.6	68.5 ± 9.7	68.6 ± 9.3	0.661
Male, *n* (%)	1,629 (48.5)	874 (40.7)	755 (62.2)	<0.001
BMI, kg/m^2^	32.1 ± 7.0	32.2 ± 7.2	31.9 ± 6.5	0.213
BMI category				0.068
Normal weight	418 (12.4)	283 (13.2)	135 (11.1)	
Overweight	1,070 (31.8)	670 (31.2)	400 (32.9)	
Class I obesity	942 (28.0)	581 (27.1)	361 (29.7)	
Class II obesity	506 (15.1)	323 (15.1)	183 (15.1)	
Class III obesity	424 (12.6)	289 (13.5)	135 (11.1)	
Waist circumference, cm	105.2 ± 16.7	104.5 ± 16.7	106.4 ± 16.6	0.002
Height, cm	167.1 ± 10.1	166.3 ± 10.1	168.6 ± 9.9	<0.001
Race category, *n* (%)				<0.001
White	2,999 (89.3)	1,880 (87.6)	1,119 (92.2)	
Black	283 (8.4)	215 (10.0)	68 (5.6)	
All others	78 (2.3)	51 (2.4)	27 (2.2)	
Heart rate, bpm	69.0 ± 10.4	70.0 ± 10.5	67.3 ± 9.9	<0.001
SBP, mmHg	129.2 ± 13.8	130.3 ± 13.7	127.4 ± 13.9	<0.001
DBP, mmHg	75.8 ± 10.7	77.0 ± 10.5	73.7 ± 10.6	<0.001
LVEF, %	59.6 ± 8.0	60.4 ± 8.0	58.4 ± 8.0	<0.001
NYHA functional class, *n* (%)				0.003
I–II	2,253 (67.1)	1,478 (68.9)	775 (63.8)	
III–IV	1,107 (32.9)	668 (31.1)	439 (36.2)	
Alcohol, drinks/week				0.583
0	2,619 (78.0)	1,678 (78.2)	941 (77.6)	
1–5	566 (16.9)	365 (17.0)	201 (16.6)	
5–10	122 (3.6)	72 (3.4)	50 (4.1)	
11+	51 (1.5)	30 (1.4)	21 (1.7)	
Current smoker, *n* (%)	345 (10.3)	195 (9.1)	150 (12.4)	0.003
**Co-morbidities, *n* (%)**				
Previous HF hospitalization	2,439 (72.6)	1,601 (74.6)	838 (69.0)	<0.001
Previous MI	877 (26.1)	–	877 (72.2)	<0.001
PCI	490 (14.6)	–	490 (40.4)	<0.001
CABG	435 (12.9)	–	435 (35.8)	<0.001
Peripheral arterial disease	313 (9.3)	110 (5.1)	203 (16.7)	<0.001
Previous stroke	260 (7.7)	129 (6.0)	131 (10.8)	<0.001
Dyslipidemia	2,028 (60.4)	1,053 (49.1)	975 (80.3)	<0.001
COPD	391 (11.6)	223 (10.4)	168 (13.8)	0.003
Hypertension	3,075 (91.5)	1,951 (90.9)	1,124 (92.6)	0.108
Atrial fibrillation	1,187 (35.3)	814 (37.9)	373 (30.7)	<0.001
Diabetes	1,090 (32.4)	585 (27.3)	505 (41.6)	<0.001
**Laboratory values**				
Serum creatinine, mg/dL	1.1 ± 0.3	1.1 ± 0.3	1.1 ± 0.3	<0.001
eGFR, mL/min × 1.73 m^2^	65.4 (25.3)	66.2 (24.2)	63.5 (26.6)	0.004
NT-proBNP, pg/ml (*N* = 508)	978.50 (1,281.25)	1,049.00 (1,282.75)	911.50 (1,238.75)	0.171
		Non-ischemic heart failure (*N* = 2,146)	Ischemic heart failure (*N* = 1,214)	
**Medications, *n* (%)**				
Diuretics	2,753 (81.9)	1,820 (84.8)	933 (76.9)	<0.001
β-Blocker	2,615 (77.8)	1,611 (75.1)	1,004 (82.7)	<0.001
Statin	1,762 (52.4)	866 (40.4)	896 (73.8)	<0.001
ACEI/ARB	2,838 (84.5)	1,820 (84.8)	1,018 (83.9)	0.494
CCB	1,266 (37.7)	820 (38.2)	446 (36.7)	0.418
Warfarin	768 (22.9)	521 (24.3)	247 (20.3)	0.010
Aspirin	2,207 (65.7)	1,246 (58.1)	961 (79.2)	<0.001

Continuous variables were presented as Mean ± SD or median (inter-quartile range). Catergorized variables were as frequency (%).

*P-value represents comparison between groups by the Student’s t-test for continuous variables and the Chi-square test for categorical variables.

HFpEF, heart failure with preserved ejection fraction; DM, diabetes mellitus; BMI, body mass index; bpm, beat per minute; SBP, systolic blood pressure; DBP, diastolic blood pressure; MI, myocardial infarction; PCI, percutaneous coronary intervention; CABG, coronary artery bypass grafting; COPD, chronic obstructive pulmonary disease; eGFR, estimated glomerular filtration rate; LVEF, left ventricular ejection fraction; NYHA, New York Heart Association; ACEI, angiotensin, converting enzyme inhibitor; ARB, angiotensin receptor blocker; CCB, calcium channel blocker.

### Association between baseline body mass index category and long-term outcomes

**FIGURE 1:**
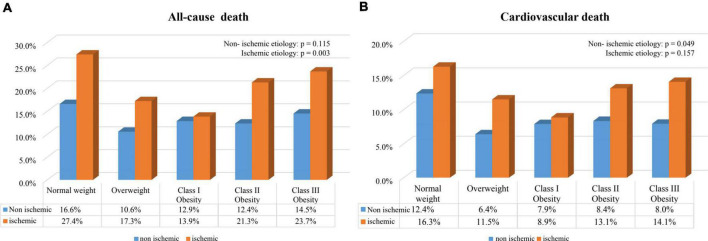
Crude death rates in HFpEF patients with non-ischemic and ischemic etiology according to baseline BMI categories. **(A)** All-cause death; **(B)** cardiovascular death.

**FIGURE 2:**
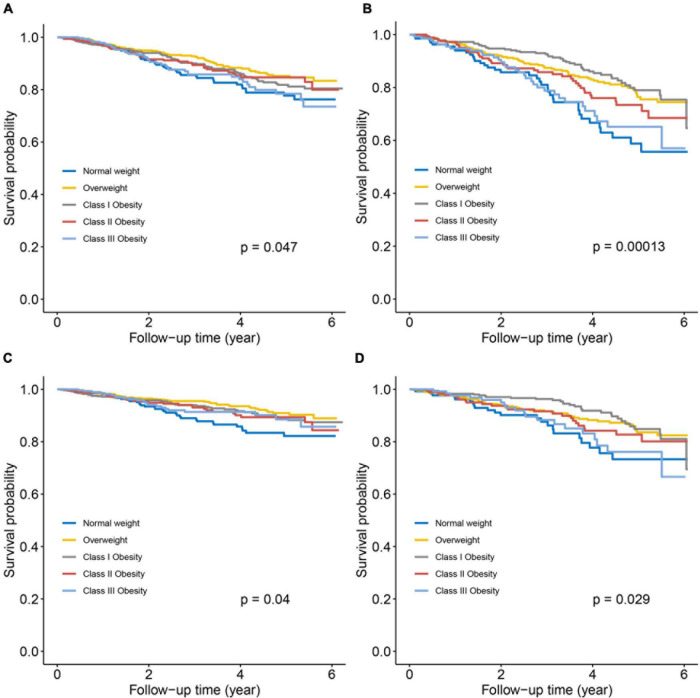
Kaplan–Meier survival curves for all-cause death **(A,B)** and cardiovascular death **(C,D)** according to baseline BMI categories in HFpEF patients with non-ischemic and ischemic etiology. **(A,C)** Non-ischemic HFpEF; **(B,D)** ischemic HFpEF.

During a median follow-up of 3.4 years, a total of 502 subjects (14.9%) died, of whom 317 were adjudicated as CV death. There were 174 (8.1%) and 143 (11.8%) CV deaths in the non-ischemic and ischemic subgroups, respectively. Crude death rate and Kaplan–Meier survival curves for all-cause death and CV death across all baseline BMI categories are shown in Figures 1, 2. Significant survival difference among all baseline BMI categories were observed both in non-ischemic and ischemic subgroups (all log-rank *p* < 0.05). After adjustment for multiple clinical covariates, compared with normal-weight, overweight and obesity remained to be associated with lower risk of long-term all-cause death in patients with non-ischemic HFpEF, with the lowest risk in class III obesity category (adjusted HR: 0.61, 95% CI 0.38–0.97, *p* = 0.038) (Table 2). In patients with ischemic HFpEF, as obesity advanced, this paradoxical relationship was gradually attenuated and even disappeared in class III obesity (adjusted HR: 0.93, 95% CI 0.56–1.57, *p* = 0.798), with the lowest risk of all-cause death in class I obesity category (adjusted HR: 0.49, 95% CI 0.32–0.76, *p* = 0.001). There was no significant interaction between BMI and HF etiology. Similar association patterns were observed with CV death. The paradoxical survival benefits were continually observed in all of the overweight and obesity categories in non-ischemic HFpEF, but disappeared in class III obesity in ischemic HFpEF (adjusted HR: 0.97, 95% CI 0.51–1.83, *p* = 0.916). Likewise, the nadir of CV death risk occurred in class III obesity (adjusted HR: 0.45, 95% CI 0.25–0.82, *p* = 0.008) in non-ischemic HFpEF, but in class I obesity (adjusted HR: 0.49, 95% CI 0.28–0.85, *p* = 0.01) in ischemic HFpEF.

**TABLE 2:** Association between baseline BMI category and long-term death according to heart failure etiology.

Outcome	Non-ischemic heart failure (*N* = 2,146)					Ischemic heart failure (*N* = 1,214)					*p* for interaction
			
	Incidence rates, per 100 person-years	Unadjusted HR (95% CI)	*P*-value	Adjusted HR* (95% CI)	*P*-value	Incidence rates, per 100 person-years	Unadjusted HR (95% CI)	*P*-value	Adjusted HR* (95% CI)	*P*-value	
All-cause death											0.858
Normal weight	4.9 (3.6–6.5)	1.00 (reference)		1.00 (reference)		8.9 (6.3–12.3)	1.00 (reference)		1.00 (reference)		
Overweight	3.0 (2.3–3.8)	0.61 (0.42–0.88)	0.009	0.66 (0.46–0.96)	0.029	4.8 (3.7–6.1)	0.51 (0.34–0.76)	0.001	0.64 (0.43–0.97)	0.033	
Class I obesity	3.7 (2.9–4.7)	0.76 (0.53–1.09)	0.134	0.62 (0.42–0.9)	0.013	3.9 (2.9–5.2)	0.43 (0.28–0.65)	<0.001	0.49 (0.32–0.76)	0.001	
Class II obesity	3.6 (2.6–5.0)	0.74 (0.49–1.13)	0.168	0.62 (0.4–0.96)	0.032	6.1 (4.3–8.3)	0.66 (0.42–1.03)	0.067	0.69 (0.43–1.1)	0.12	
Class III obesity	4.8 (3.4–6.5)	0.98 (0.65–1.49)	0.931	0.61 (0.38–0.97)	0.038	7.8 (5.4–11.1)	0.89 (0.55–1.42)	0.615	0.93 (0.56–1.57)	0.798	
Cardiovascular death											0.652
Normal weight	3.7 (2.5–5.1)	1.00 (reference)		1.00 (reference)		5.3 (3.3–8.1)	1.00 (reference)		1.00 (reference)		
Overweight	1.8 (1.3–2.4)	0.50 (0.32–0.78)	0.002	0.55 (0.35–0.86)	0.01	3.2 (2.3–4.3)	0.57 (0.34–0.95)	0.032	0.63 (0.38–1.06)	0.08	
Class I obesity	2.3 (1.7–3.0)	0.62 (0.4–0.97)	0.035	0.53 (0.34–0.84)	0.007	2.5 (1.7–3.6)	0.46 (0.27–0.79)	0.005	0.49 (0.28–0.85)	0.01	
Class II obesity	2.5 (1.6–3.6)	0.68 (0.41–1.12)	0.126	0.58 (0.34–0.99)	0.044	3.7 (2.4–5.6)	0.68 (0.38–1.21)	0.191	0.66 (0.37–1.2)	0.171	
Class III obesity	2.6 (1.7–3.9)	0.72 (0.42–1.22)	0.22	0.45 (0.25–0.82)	0.008	4.7 (2.8–7.3)	0.89 (0.48–1.65)	0.714	0.97 (0.51–1.83)	0.916	

*Adjusted for age, sex, race, randomization group, region of enrollment (Russia/Georgia versus the Americas), NYHA functional class, heart rate, systolic blood pressure, DM, stroke, atrial fibrillation, serum creatinine, and weight change.

### Association between continuous variable of baseline body mass index and long-term outcomes

**FIGURE 3:**
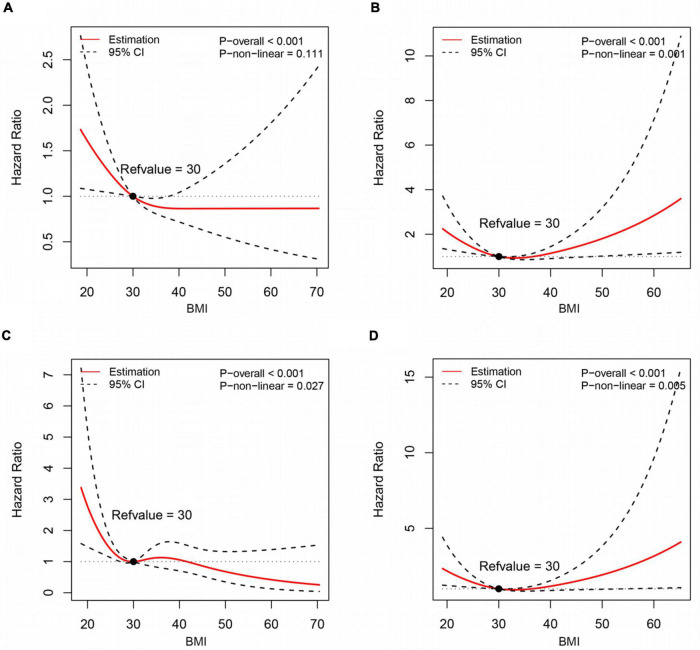
Association of baseline BMI with long-term all-cause death **(A,B)** and cardiovascular death **(C,D)** in HFpEF patients according to HF etiology, using restricted cubic spline models. **(A,C)** Non-ischemic HFpEF; **(B,D)** ischemic HFpEF.

Figure 3 showed the flexible relationships between baseline BMI and long-term mortality according to HF etiology with smooth spline plots. There was evidence of paradoxical survival benefit both in non-ischemic HFpEF and ischemic HFpEF, with different patterns seen: for non-ischemic subgroup, steep decrease in all-cause death risk was observed with a BMI lower than 30 kg/m^2^, and continue to decrease gently with a BMI higher than 30 kg/m^2^ (Figure 3A); for ischemic subgroup, the hazard of all-cause death was consistently attenuated with a BMI lower than 30 kg/m^2^, but increase gradually with a BMI higher than 30 kg/m^2^, with a J-shaped relationship observed (Figure 3B).

Analyses for CV death demonstrated qualitatively similar findings as all-cause death (Figures 3C,D). There was evidence of non-linearity for BMI and CV death, irrespective of HF etiology. For non-ischemic HFpEF subgroup, a marked decrease in CV death risk was observed with a BMI lower than 30 kg/m^2^, but minimal elevation in risk with a BMI between 30 and 40 kg/m^2^. With a BMI above 40 kg/m^2^, CV death risk tended to decrease again (Figure 3C). While for ischemic subgroup, similar J-shaped relationship was observed as the all-cause death (Figure 3D).

### Association between time-updated body mass index category and short-term outcomes

Estimated associations between time-updated BMI category and all-cause death and CV death in the short-term follow-up are shown in Table 3. In multivariate analyses, the beneficial effects of overweight and obesity on short-term survival were consistently observed in all BMI categories, with similar nadirs for all-cause death risk at class III obesity category both in HFpEF with non-ischemic etiology (adjusted HR: 0.40, 95% CI 0.16–1.04) and ischemic etiology (adjusted HR: 0.48, 95% CI 0.27–0.86). Regarding the CV death, the lowest risk occurred in overweight category (adjusted HR: 0.66, 95% CI 0.54–0.81) in non-ischemic HFpEF, and overweight/class I obesity categories (both adjusted HR: 0.71, 95% CI 0.58–0.86) in ischemic HFpEF, respectively. Likewise, there was no significant interaction between HF etiology and time-updated BMI for all-cause death and CV death.

**TABLE 3:** Association between time-updated BMI category and short-term death according to heart failure etiology.

Outcome	Non-ischemic heart failure				Ischemic heart failure				*p* for interaction
			
	Unadjusted HR (95% CI)	*P*-value	Adjusted HR* (95% CI)	*P*-value	Unadjusted HR (95% CI)		Adjusted HR* (95% CI)	*P*-value	
**All-cause death**									0.167
Normal weight	1.00 (reference)		1.00 (reference)		1.00 (reference)		1.00 (reference)		
Overweight	0.56 (0.39–0.8)	0.001	0.62 (0.42–0.93)	0.02	0.43 (0.29–0.63)	<0.001	0.52 (0.35–0.77)	0.001	
Class I obesity	0.64 (0.44–0.92)	0.015	0.59 (0.36–0.97)	0.039	0.51 (0.34–0.74)	0.001	0.58 (0.39–0.85)	0.006	
Class II obesity	0.90 (0.61–1.33)	0.598	0.65 (0.34–1.24)	0.189	0.63 (0.41–0.97)	0.036	0.67 (0.43–1.04)	0.076	
Class III obesity	0.75 (0.49–1.16)	0.202	0.40 (0.16–1.04)	0.06	0.53 (0.31–0.9)	0.019	0.48 (0.27–0.86)	0.014	
**Cardiovascular death**									0.846
Normal weight	1.00 (reference)		1.00 (reference)		1.00 (reference)		1.00 (reference)		
Overweight	0.55 (0.46–0.65)	<0.001	0.66 (0.54–0.81)	<0.001	0.63 (0.52–0.76)	<0.001	0.71 (0.58–0.86)	<0.001	
Class I obesity	0.59 (0.49–0.7)	<0.001	0.72 (0.55–0.95)	0.018	0.63 (0.52–0.77)	<0.001	0.71 (0.58–0.86)	0.001	
Class II obesity	0.75 (0.62–0.92)	0.005	0.93 (0.65–1.33)	0.683	0.71 (0.57–0.88)	0.002	0.77 (0.61–0.97)	0.027	
Class III obesity	0.56 (0.45–0.7)	<0.001	0.78 (0.46–1.33)	0.366	0.76 (0.59–0.97)	0.027	0.80 (0.61–1.05)	0.103	

*Adjusted for age, sex, race, randomization group, region of enrollment (Russia/Georgia versus the Americas), time-update NYHA functional class, time-update heart rate, time-update systolic blood pressure, DM, stroke, atrial fibrillation, serum creatinine, and baseline BMI.

### Sensitivity analyses

Sensitivity analysis using the Americas HFpEF of the TOPCAT trial demonstrated broadly similar results as the total cohort (Supplementary Tables 1, 2 and Supplementary Figures 1–3). Overweight and class I/II obesity defined with baseline BMI value were associated with reduced risk of long-term all-cause death both in non-ischemic HFpEF and ischemic HFpEF (Supplementary Table 1). However, class III obesity conferred survival benefit only in non-ischemic HFpEF (adjusted HR: 0.45, 95% CI 0.29–0.71, *p* = 0.001) but not in ischemic HFpEF (adjusted HR: 1.08, 95% CI 0.61–1.93, *p* = 0.791). Smooth spline plots demonstrate similar inverse relationship of baseline BMI and long-term death in non-ischemic and ischemic HFpEF with a BMI lower than 30 kg/m^2^. However, at BMI higher than 30 kg/m^2^, the inverse relationship was limited to the non-ischemic subgroup (Supplementary Figure 3). When modeling for time-updated BMI, overweight and all obesity categories were associated with decreased risk of short-term all-cause death both in non-ischemic and ischemic HFpEF (Supplementary Table 2).

## Discussion

In this retrospective cohort study of HFpEF patients, the “obesity paradox” was observed both in patients with non-ischemic and ischemic etiology. Overweight or class I–III obesity was associated with progressively lower risk of death in the long-term follow-up in non-ischemic HFpEF. However, the long-term survival benefits of obesity were gradually attenuated and even disappeared in class III obesity category in ischemic HFpEF. Although there was similar inverse association between baseline BMI and death in non-ischemic and ischemic HFpEF at BMI lower than 30 kg/m^2^, at BMI higher than 30 kg/m^2^ the inverse association was confined to those with non-ischemic HFpEF. In the short-term follow-up, overweight and class I–III obesity were continually associated with better survival both in non-ischemic and ischemic HFpEF, with the same lowest death risk at class III obesity for all-cause death and at overweight/class I obesity for CV death.

The existence of a paradoxical effect of obesity on death has been a topic of debate in the HF studies. A randomized controlled trial of 7,599 patients with symptomatic HFpEF and HFrEF (19) demonstrated that the risk of death was gradually decreased with an increase of BMI, irrespective of LVEF. Similar finding was confirmed by several meta-analyses (4, 20–22), which demonstrated an inverse or U-shaped relationship between BMI and mortality in HF. Subsequently, plenty studies have come up with several potential confounders in the “obesity paradox” in HF population, such as age (23), gender (24), diabetes (25) and HF etiology (9, 10). A prior study by Arena et al. (8), which enrolled 1,160 patients with HFrEF, demonstrated that obesity conferred improved survival in patients with HFrEF, irrespective of HF etiology. In contrast, Zamora et al. (9) analyzed 504 HF patients (mainly with LVEF <40%) and found that obesity-related survival benefit was confined to those with non-ischemic HF. However, both of the two studies did not examine the effect of different obesity grades on prognosis in patients with HF according to HF etiology. More recently, Gentile et al. (10) evaluated 5,155 HF patients with a broad spectrum of LVEF (included 982 patients with HFpEF) and stratified patients according to the latest WHO BMI classifications. It was also observed that obesity-related survival benefits only existed in non-ischemic HF. In the present study, we conduct a dedicated analysis of a larger sample of HFpEF patients, finding that overweight and obesity were associated with better survival both in non-ischemic and ischemic HFpEF. Several possible explanations for the obesity paradox in HF have been raised, including the inverse relationship between NT-proBNP levels and BMI (26), attenuated response to the renin-angiotensin-aldosterone system (27), and increased muscle mass and muscular strength in those with high BMI (3). The discrepancy between our results and those previously published studies may be due to the difference in sample size and clinical characteristic of the study populations, as well as the heterogeneity of BMI classifications. In the study by Gentile et al. (10), the patients enrolled were mostly males, with relatively lower BMI level and less burden of co-morbidities, which may also modify the relationship of BMI and outcomes in patients with HF.

Notably, although there was a similarly inverse relationship of overweight with long-term death in non-ischemic and ischemic patients, there were a differential effect of obesity on long-term death according to the severity of obesity between the two subgroups. In non-ischemic HFpEF patients, class I–III obesity were associated with progressively lower risk of long-term death. However, in ischemic subgroup, the obesity-related survival benefit was gradually attenuated and even disappeared in patients with class III obesity in the long-term follow-up. Smooth spline plots further confirmed the different associations of BMI and long-term death at BMI higher than 30 kg/m^2^ in non-ischemic versus ischemic patients. As the burden of comorbidities in non-ischemic HFpEF was significantly lower than that in ischemic HFpEF (as shown in Table 1), the progressive catabolic state that underlies the pathophysiology of HF may play a predominant role in the prognosis of non-ischemic patients. In this case, obesity might exert a continually protective effect in non-ischemic HFpEF patients. However, in patients with ischemic HFpEF, the relationship of obesity with prognosis could be modified by atherosclerotic risk and high burden of comorbidities, which is associated with a greater likelihood of cardiovascular complications and death. A 10 years retrospective study by Pan et al. (28), which enrolled patients undergoing primary coronary artery bypass surgery (CABG) (*n* = 9,862), demonstrated that obesity was not associated with increased morbidity or mortality, however, patients with BMI >40 kg/m^2^ was independently associated with increased risks of adverse postoperative outcomes. A prior meta-analysis by Sharma et al. (29), which included 26 percutaneous coronary intervention (PCI) studies with more than 189,000 patients and 12 CABG studies with more than 60,000 patients, found that underweight patients had the highest risk but overweight patients had the lowest risk of all-cause death over an average follow-up of 1.7 years, as compared with those with normal BMI. However, severely obese patients had a significantly higher risk of cardiac mortality after CABG, which was in line with those reported by Pan et al. (28). Consistent with these previous reports, our study demonstrated a long-term survival benefit in ischemic HFpEF patients with overweight and class I/II obesity, however, not in those with class III obesity.

Over recent decades, numerous studies have evidenced an obesity paradox in patients with coronary artery disease (CAD) or after coronary revascularization (29–33). Hence, it was plausible to find beneficial effect of overweight/obesity on survival in HFpEF with ischemic etiology in the present study. In addition, our study extends previous evidence by demonstrating a different effect of class III obesity on survival between the short-term and long-term follow-up in patients with ischemic HFpEF. Dhoot et al. reviewed 413,673 patients with acute myocardial infarction and found that patients with morbid obesity (BMI ≥40 kg/m^2^) had lower risk of in-hospital mortality, compared with those not morbidly obese (34). Subsequently, a large meta-analysis by Wang et al. (32), which pooled 89 studies with 1,300,794 patients with CAD, found that obesity paradox was evident during short-term follow-up even in patients with class III obesity. However, during long-term follow-up, those with CAD and moderate/severe obesity (class II–III obesity) experienced a higher mortality. Similar findings were also reported in studies by Lavie et al. (35). In line with these previous findings, our results showed that class III obesity was beneficial for short-term survival but not for long-term survival in patients with ischemic HFpEF. These consistent finding suggested that the obesity paradox in patients with ischemic HFpEF might be time-dependent. In fact, extremely obese patients are more likely to receive more aggressive treatment (36) and tolerate more cardioprotective medications (37), which could be protective for the short-term survival. Nevertheless, morbidly obese HF patients had markedly increased cardiac structural and functional abnormalities (38), which were known as risk factors for increased ventricular arrhythmias and sudden cardiac death. In addition, class III obesity has been described as a systemic inflammatory and prothrombotic state (39), which may also contribute to adverse prognosis. Therefore, over a long-term follow-up, the obesity-related benefit may be largely offset by the worse overall CV risk factor profiles and higher burden of co-morbidities in ischemic patients with extremely obesity. Studies by Lin et al. (40) and Li et al. (41) consistently demonstrated a J-shaped relationship between BMI and mortality for longer than a 5-year follow-up in patients with CAD. It was speculated that the natural course of an elevated risk of re-stenosis in grafting vessels (42) during the long-term follow-up and the increased risk for thrombosis after PCI with bare-metal or drug-eluting stents in extremely obese patients (43, 44) may contribute to the long-term increased mortality risk in patients with CAD and obesity.

It was not completely known why the lowest risk of short-term mortality occurred in different obesity categories between all-cause death and CV death, with the former in class III obesity and the latter in overweight/class I obesity, irrespective of HF etiology. A possible explanation is that obesity may be a sign of less preexisting illness or underlying chronic non-cardiovascular disease, thus favorable for the overall survival. However, as obesity advanced, the prevalence of CV risk factors and CVD markedly increased (45). In this case, the protective effect of obesity on CV death may be gradually attenuated across increasing obesity severity. This speculation was also supported by data from Jerant et al. (46), which showed that an association of severe obesity with increased mortality was attributable to coexisting diabetes and hypertension.

## Strengths and limitations

The major strengths of our study are that we extend previous observations by demonstrating the impact of ischemic etiology on the association of BMI and mortality in HFpEF patients during the short-term and long-term follow-up, respectively. Specifically, overweight and obesity-related survival benefit were both evident in non-ischemic and ischemic HFpEF patients in the short-term follow-up. However, we found the survival benefit of class III obesity in ischemic heart failure group was time-dependent, which was present in the short-term follow-up but disappeared during the long-term follow-up. A possible explanation for this decrescendo effect might be related to the underlying atherosclerotic risk and high burden of comorbidities, as well as the increased risk for thrombosis after PCI or re-stenosis in grafting vessels after CABG in severe obese patients with ischemic HF. All of these conditions were associated with a greater likelihood of cardiovascular death, and the prognosis of patients during the long-term follow-up duration are more likely to be affected by this problem.

Our study has several potential limitations as follows. First, the ability of BMI to evaluate the distribution and degree of adiposity appeared to be limited; also, BMI is unable to differentiate between body fat and lean mass. Therefore, we could not determine the interplay of fat-but-fit paradigm and HF etiology in the study population. Second, given that the prevalence of most diseases is increased in older people, and data about weight changes prior to the trial enrollment is not available, we could not eliminate the risk of reverse-causation bias. However, our study took into account weight change during the follow-up when analyzing the long-term effect of various BMI categories on death, and controlled for baseline BMI when analyzing the short-term effect of various BMI categories on death, which may help to reduce the susceptibility to reverse-causation bias. Third, the number of underweight subjects is relatively small and were not included in the analysis, potentially limiting the extrapolation of our findings to the general population of HFpEF patients. We did not adjust all CV risk factors (such as LVEF and brain natriuretic peptide) in the multivariate analyses because of the relatively limited data, thus could not rule out the possibility of residual confounding effects in our analysis. Likewise, we could not perform multivariate adjusted cox regression analysis to elucidate the impact of ischemic etiology on the association of five BMI categories and mortality in patients with EF higher than 50% or different levels of natriuretic peptides because of the relatively small sample size of patients with available EF or brain natriuretic peptide data. Finally, although we did not observe a protective effect of class III obesity on long-term death in ischemic HFpEF patients, it is not clear whether targeted weight loss in morbidly obese patients is beneficial or not. Large randomized control trials evaluating the impact of intentional weight reduction or weight gain in various BMI categories may help to resolve this issue.

## Conclusion

Overweight and obesity defined by BMI confer survival benefits both in non-ischemic and ischemic HFpEF patients. This paradoxical relationship persists in all BMI categories in patients with non-ischemic HFpEF. However, in patients with ischemic HFpEF, although an obesity paradox was noted during short-term follow-up for all BMI categories, class III obesity was not associated with better survival during long-term follow-up.

## Data availability statement

The datasets presented in this study can be found in online repositories. The names of the repository/repositories and accession number(s) can be found in the article/Supplementary material.

## Ethics statement

The patients/participants provided their written informed consent to participate in this study. Written informed consent was obtained from the individual(s) for the publication of any potentially identifiable images or data included in this article.

## Author contributions

All authors listed have made a substantial, direct, and intellectual contribution to the work, and approved it for publication.
