# Supplementary material for: Associations of body mass index with mortality in heart failure with preserved ejection fraction patients with ischemic versus non-ischemic etiology
Source: Front Cardiovasc Med. 2022 Aug 4;9:966745. doi: 10.3389/fcvm.2022.966745 (PMC9386382; doi:10.3389/fcvm.2022.966745)
Supplement: Supplementary file 1 [file Data_Sheet_1.docx]

**Table S1**. Association between baseline BMI category and long-term death in American HFpEF according to HF etiology

| **Outcome** | **Non-ischemic Heart Failure (N =1066)** | | | | | **Ischemic Heart Failure (N =641)** | | | | | **p for interaction** |
| --- | --- | --- | --- | --- | --- | --- | --- | --- | --- | --- | --- |
|  | **Incidence rates, per 100 person-years** | **Unadjusted HR (95%CI)** | **p-value** | **Adjusted HR*** **(95%CI)** | **p-value** | **Incidence rates, per 100 person-years** | **Unadjusted HR (95%CI)** | **p-value** | **Adjusted HR*(95%CI)** | **p-value** |  |
| **All-cause death** |  |  |  |  |  |  |  |  |  |  | 0.214 |
| Normal weight | 11.2 (8.0-15.2) | 1.00 (Reference) |  | 1.00 (Reference) |  | 12.3 (8.0-18.2) | 1.00 (Reference) |  | 1.00 (Reference) |  |  |
| Overweight | 6.4 (4.7-8.5) | 0.56 (0.37-0.86) | 0.007 | 0.54 (0.35-0.83) | 0.005 | 8.1 (5.8-11.0) | 0.64 (0.39-1.06) | 0.081 | 0.71 (0.43-1.17) | 0.175 |  |
| Class I Obesity | 6.0 (4.4-7.8) | 0.52 (0.35-0.79) | 0.002 | 0.45 (0.29-0.7) | <0.001 | 6.3 (4.5-8.7) | 0.50 (0.3-0.83) | 0.007 | 0.59 (0.35-1) | 0.048 |  |
| Class II Obesity | 5.6 (3.8-7.9) | 0.49 (0.31-0.78) | 0.003 | 0.50 (0.3-0.81) | 0.005 | 7.7 (5.1-11.0) | 0.61 (0.36-1.04) | 0.069 | 0.78 (0.45-1.35) | 0.373 |  |
| Class III Obesity | 5.1 (3.5-7.1) | 0.45 (0.29-0.71) | 0.001 | 0.43 (0.26-0.73) | 0.002 | 8.8 (5.9-12.6) | 0.72 (0.42-1.22) | 0.222 | 1.08 (0.61-1.93) | 0.791 |  |
| **Cardiovascular death** |  |  |  |  |  |  |  |  |  |  | 0.071 |
| Normal weight | 7.9 (5.3-11.4) | 1.00 (Reference) |  | 1.00 (Reference) |  | 5.9 (3.1-10.3) | 1.00 (Reference) |  | 1.00 (Reference) |  |  |
| Overweight | 3.7 (2.4-5.4) | 0.46 (0.27-0.78) | 0.004 | 0.49 (0.29-0.84) | 0.01 | 4.4 (2.8-6.7) | 0.73 (0.36-1.47) | 0.381 | 0.76 (0.38-1.54) | 0.447 |  |
| Class I Obesity | 3.3 (2.2-4.7) | 0.41 (0.24-0.68) | 0.001 | 0.40 (0.23-0.68) | 0.001 | 3.5 (2.2-5.4) | 0.57 (0.28-1.17) | 0.127 | 0.58 (0.28-1.18) | 0.133 |  |
| Class II Obesity | 3.3 (2.0-5.2) | 0.41 (0.23-0.74) | 0.003 | 0.48 (0.26-0.88) | 0.017 | 4.5 (2.6-7.2) | 0.74 (0.35-1.55) | 0.426 | 0.70 (0.33-1.47) | 0.346 |  |
| Class III Obesity | 2.8 (1.7-4.4) | 0.35 (0.2-0.63) | <0.001 | 0.39 (0.21-0.74) | 0.004 | 4.8 (2.8-7.9) | 0.83 (0.39-1.75) | 0.623 | 0.79 (0.37-1.67) | 0.538 |  |

***** adjusted for age, sex, race, randomization group, NYHA functional class, heart rate, systolic blood pressure, DM, stoke, Atrial fibrillation, serum creatinine and weight change.

***Abbreviations:*** HFpEF, heart failure with preserved ejection fraction; HF, heart failure.

**Table S2.** Association between time-updated BMI category and short-term death in American HFpEF according to HF etiology

| **Outcome** | **Non-ischemic Heart Failure** | | | | **Ischemic Heart Failure** | | | | **p for interaction** |
| --- | --- | --- | --- | --- | --- | --- | --- | --- | --- |
|  |  |  |  |  |  |  |  |  |  |
|  | **Unadjusted HR (95%CI)** | **p-value** | **Adjusted HR*** **(95%CI)** | **p-value** | **Unadjusted HR (95%CI)** |  | **Adjusted HR*(95%CI)** | **p-value** |  |
| **All-cause death** |  |  |  |  |  |  |  |  | 0.937 |
| Normal weight | 1.00 (Reference) |  | 1.00 (Reference) |  | 1.00 (Reference) |  | 1.00 (Reference) |  |  |
| Overweight | 0.52 (0.34-0.79) | 0.002 | 0.52 (0.33-0.83) | 0.006 | 0.53 (0.33-0.85) | 0.008 | 0.53 (0.33-0.86) | 0.01 |  |
| Class I Obesity | 0.46 (0.31-0.7) | <0.001 | 0.48 (0.27-0.85) | 0.011 | 0.57 (0.36-0.9) | 0.016 | 0.63 (0.39-1) | 0.049 |  |
| Class II Obesity | 0.52 (0.33-0.81) | 0.004 | 0.50 (0.24-1.03) | 0.06 | 0.61 (0.37-1) | 0.05 | 0.72 (0.43-1.22) | 0.221 |  |
| Class III Obesity | 0.41 (0.26-0.65) | <0.001 | 0.38 (0.13-1.09) | 0.073 | 0.43 (0.24-0.77) | 0.005 | 0.53 (0.28-1.02) | 0.056 |  |
| **Cardiovascular death** |  |  |  |  |  |  |  |  | 0.008 |
| Normal weight | 1.00 (Reference) |  | 1.00 (Reference) |  | 1.00 (Reference) |  | 1.00 (Reference) |  |  |
| Overweight | 0.46 (0.37-0.57) | <0.001 | 0.53 (0.41-0.68) | <0.001 | 0.81 (0.63-1.04) | 0.105 | 0.92 (0.71-1.19) | 0.52 |  |
| Class I Obesity | 0.40 (0.32-0.5) | <0.001 | 0.55 (0.4-0.75) | <0.001 | 0.80 (0.62-1.02) | 0.076 | 0.87 (0.68-1.13) | 0.297 |  |
| Class II Obesity | 0.41 (0.32-0.51) | <0.001 | 0.62 (0.41-0.95) | 0.029 | 0.66 (0.5-0.87) | 0.004 | 0.81 (0.6-1.09) | 0.168 |  |
| Class III Obesity | 0.33 (0.26-0.42) | <0.001 | 0.67 (0.37-1.23) | 0.197 | 0.72 (0.54-0.96) | 0.026 | 0.91 (0.66-1.25) | 0.542 |  |

***** adjusted for age, sex, race, randomization group, time-update NYHA functional class, time-update heart rate, time-update systolic blood pressure, DM, stoke, Atrial fibrillation, serum creatinine and baseline BMI.

***Abbreviations:*** HFpEF, heart failure with preserved ejection fraction; HF, heart failure.


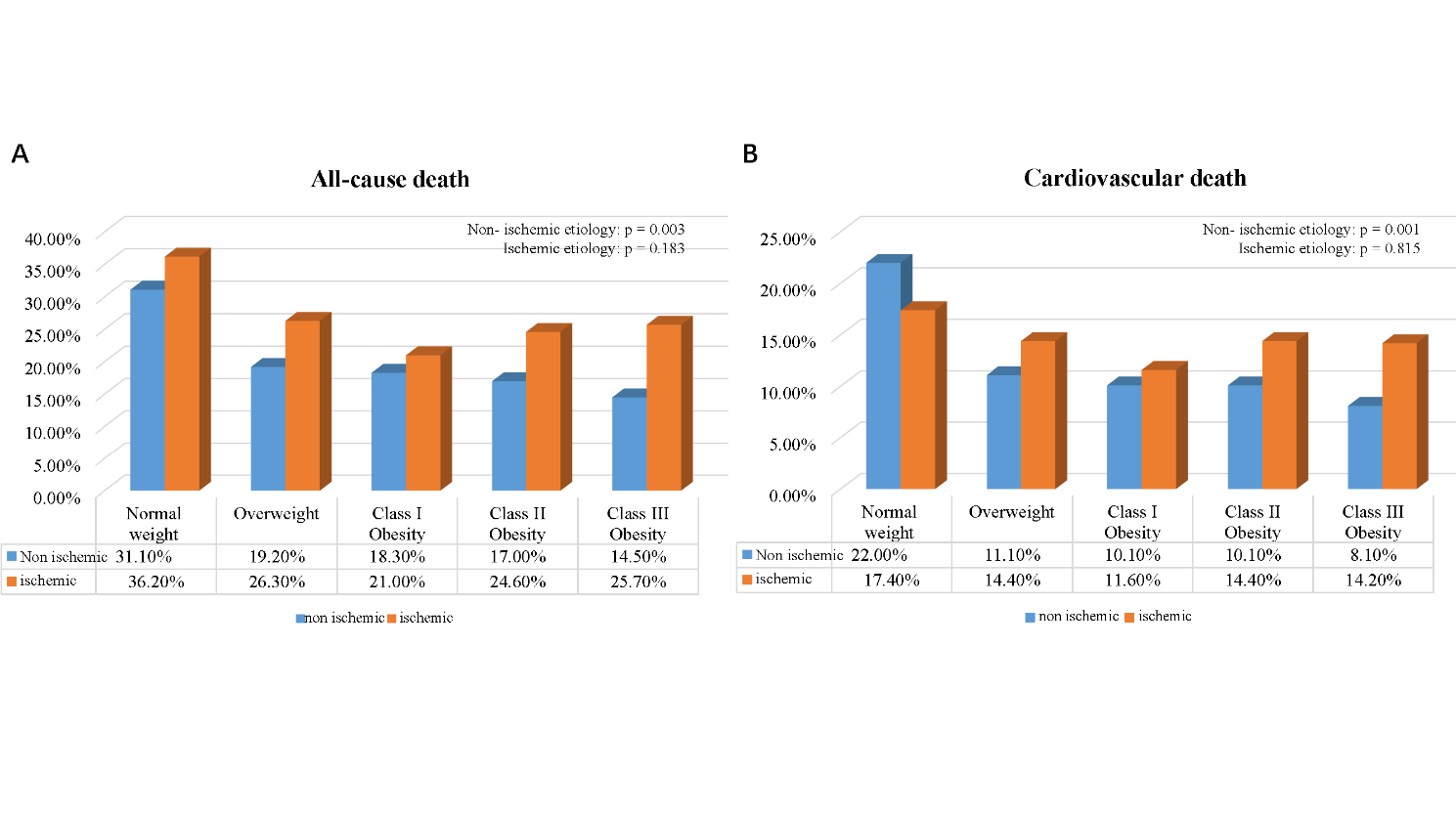


**Figure S1.** Crude death rates in American HFpEF with non-ischemic and ischemic etiology according to baseline BMI categories. (A) all-cause death; (B) cardiovascular death.


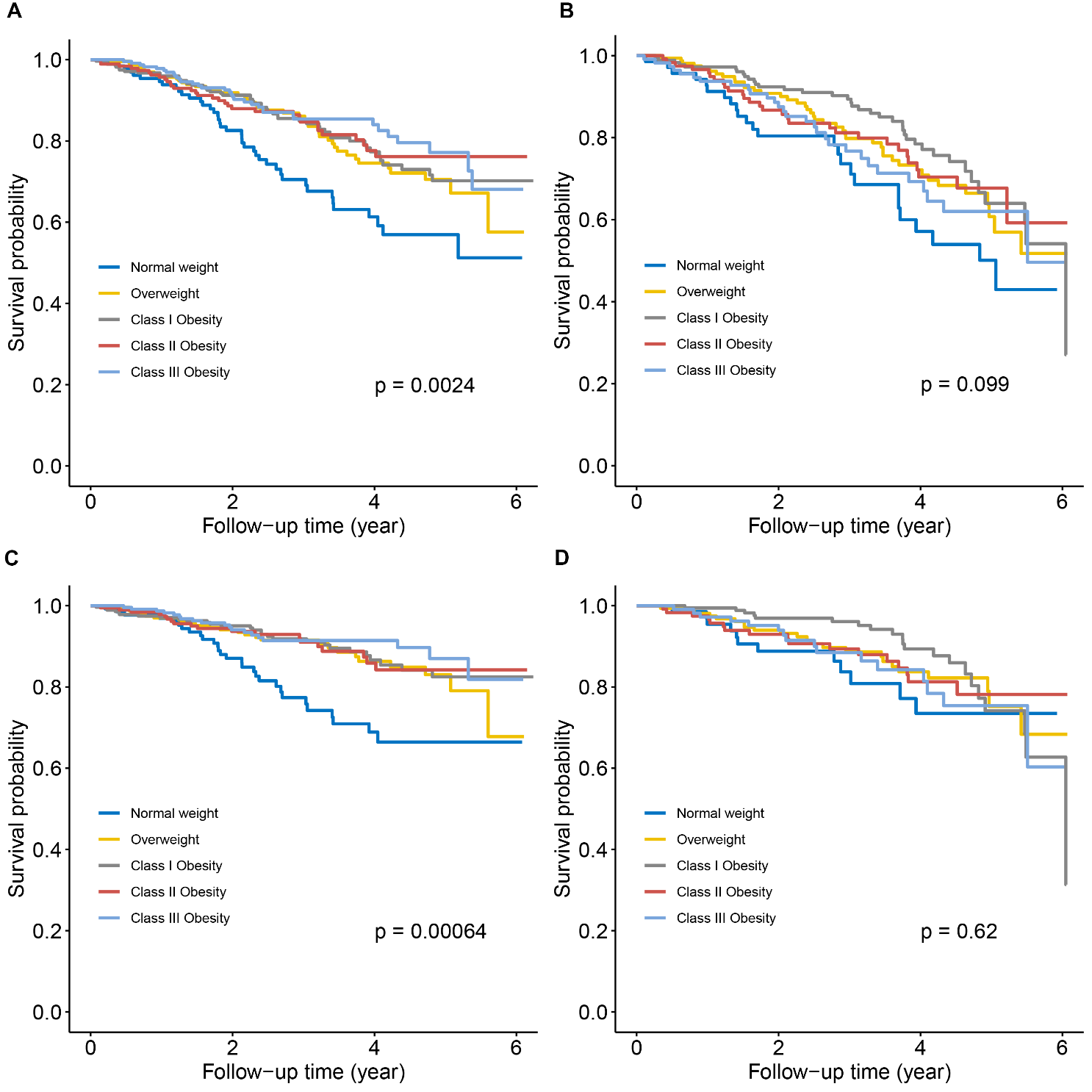


**Figure S2.** Risk for all-cause death (A, B) and cardiovascular death (C, D) by baseline BMI categories in American HFpEF patients according to HF etiology. (A, C) non-ischemic HFpEF; (B, D) ischemic HFpEF.


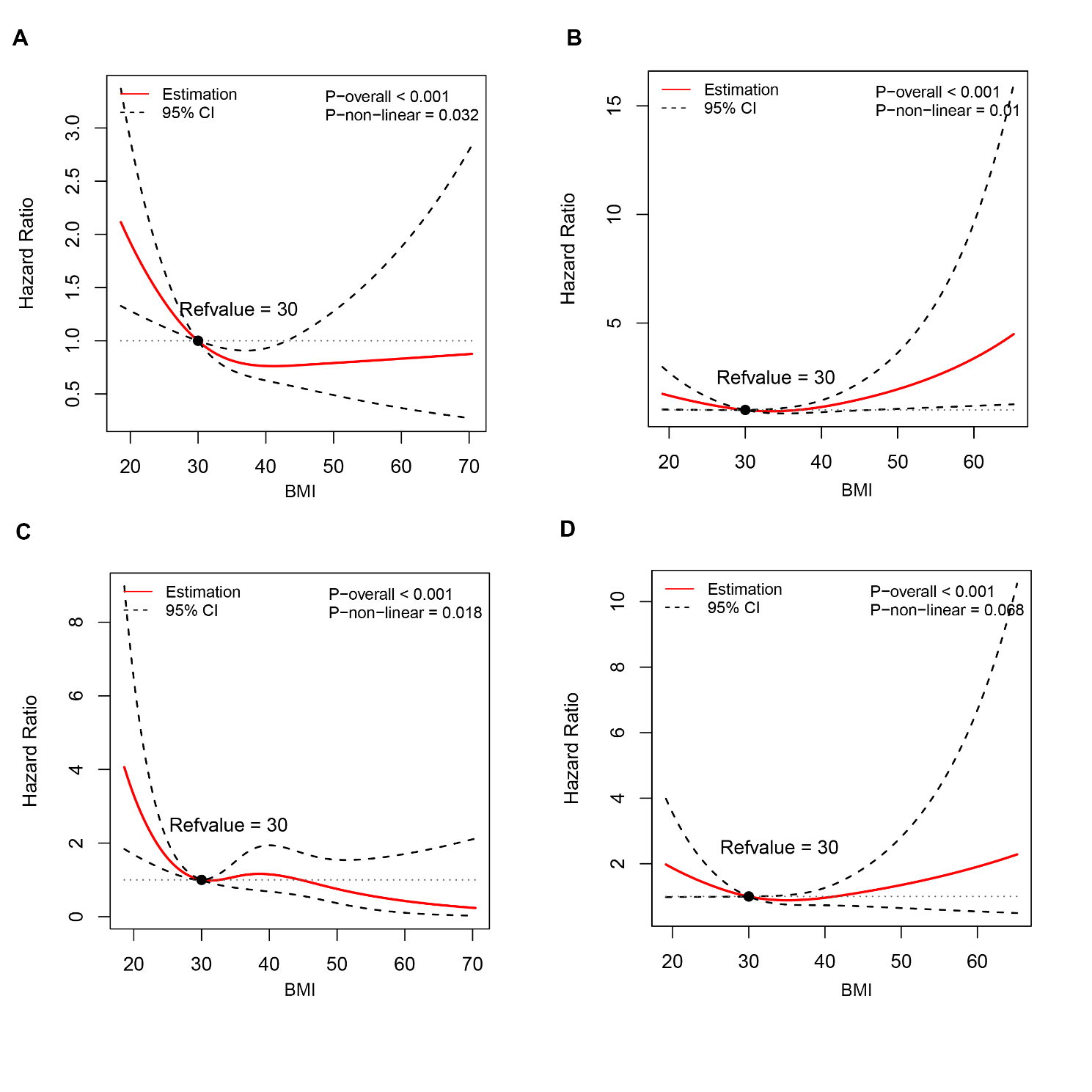


**Figure S3.** Association of baseline BMI with long-term all-cause death (A, B) and cardiovascular death (C, D) in American HFpEF patients according to HF etiology, using restricted cubic spline models. (A, C) non-ischemic HFpEF; (B, D) ischemic HFpEF.
